# Randomized Comparative Efficacy of Dexamethasone to Prevent Postextubation Upper Airway Complications in Children and Adults in ICU

**Published:** 2009-08

**Authors:** Dinesh Malhotra, Showkat Gurcoo, Shagufta Qazi, Satyadev Gupta

**Affiliations:** 1 Senior resident, Department of Anaesthesiology And Critical Care, Govt. Medical College Jammu, J&K India; 2 Associate Professor, SKIMS, Srinagar; 3 Professor, SKIMS, Srinagar; 4 Ex. Professor and head, Department of Anaesthesiology And Critical Care, Govt. Medical College Jammu, J&K India

**Keywords:** Postextubation, Complications, Laryngeal edema, Stridor, Dexamethasone

## Abstract

**Summary:**

Prophylactic steroid therapy to reduce the occurrence of postextubation laryngeal edema is controversial. Only a limited number of prospective trials involve adults and children in an intensive care unit. The purpose of this study was to ascertain whether administration of multiple doses of dexamethasone to critically ill, intubated patients reduces or prevents the occurrence of postextubation laryngeal edema / stridor and its risk factors. Another specific objective of our study was to investigate whether an after-effect (that is, a transient lingering benefit) exists 24 hours after the discontinuation of dexamethasone In a prospective, randomized, double-blind control study, a total of 120 patients were randomly allocated both in children and adult population, who were ventilated more than 24 hours in ICU; into study and Control group. Study group comprising 60 patients with 30adults and 30 children. Study group adults received 8mg dexamethasone 4 doses i.e 4 hours prior to planned extubation, at extubation and 6 and 12 hours after extubation. Children received 0.5 mg.Kg^−1^ dose with maximum of 8mg at similar intervals. Control group comprising of 30 adults and 30 children who received placebo or saline at similar intervals. There was statistically significant difference (p = 0.019) in comparison of failed extubation (those who cannot withstand extubation and reintubated) in children with respect to adults. Moreover, duration of intubation (*p* =0.014) and female gender were also risk factors for failed extubation. We concluded that prophylactic use of intravenous dexamethasone is useful in preventing postextubation laryngeal edema/stridor in children but not in adults.

## Introduction

Endotracheal intubation is a routine maneuver in Intensive Care Unit used to provide a patent airway and various modes of positive pressure ventilation in critically ill patients.^1^ Laryngotracheal injury related to intubation may cause narrowing of the airway due to edema of the glottis. Laryngeal edema is more common after endotracheal intubation for more than 36 hours^1^. Edema inthis region is associated with the increased risks for postextubation stridor, which increase reintubation rate, aprocess referred to as failed extubation.

Reintubation may result in morbidity and mortality^2^‐^6^. Prolonged intubation or excessive endotracheal tube cuffpres sure can initiate mucosal erosion and cartilage necrosis followed bytracheal stenosis.^2^ Mortality associated with reintubation has been estimated to be as high as 30% to 40%^4^,^6^. Because the presence of an endotracheal tube (ETT) precludes direct visualization of the upper airway, recognition of the edema due to laryngotracheal injury is often difficult be as high as 30% to 40%^4^,^6^ The prevalence of postextubation stridor ranges between 6% and 37% in intubated patients^7^‐^12^

Factors that may increase the likelihood of air-way damage include repeated passage of endotracheal tube, prolonged intubation for more than 24-hours and alarge size of endotrachealtube in relation to the size of the glottis, low Glasgow Coma Scale score, and fe-male sex. Female sex is at ahigher risk of developing complications probably because ofsmaller size of larynx in comparison to males.^13^,^14^ Mucosalmembrane in males tends to be more resistant to trauma.^15^ Cervical flexion and extension, inspiration, cough and deglutination can all affect the relative position of larynxand endotrachealtube. Dynamic interactions may cause injury eitherby alternations in pressure on the mucosa orby frictional erosion^16^. Radiographic examination has shown that endotracheal tube can move 3.8 cm when head is moved from flexionto extension^17^.

Laryngeal edema is most commonly symptomatic in children because of their small airway size, more severely reduced in cross-sectional area by edema. Reactive subglottic edema in children at the cricoid ring can leadto post extubation stridor.^18^ Laryngeal edema has been reported after tracheal extubation as one of the serious complications and causes significant morbidity as well as prolongs the stay in ICU. Pre-extubation dexamethas one therapy could prevent the complications of pro longed intubation which becomes obvious at the time of extubation. By reducing the degree of laryngeal edema, corticosteroids may reduce the incidence of reintubation or failed extubation, which has clinical, economical and ethical problems in patients admitted in ICU.^9^,^10^

Controversy still exists regarding the effectiveness ofprophylactic steroid therapy for patients at risk for postextubation stridor^19^‐^22^. Some studies involving postextubation stridor and analyses of outcomes for those receiving steroids during intubation have yielded inconclusive ornegative results^19^. Only alimited number of randomized trials involving adults and evaluating the benefits of cortico steroid therapy prior to extuba-tion have been conducted^21^,^22^. Moreover, studies regarding the efficacy of prophylactic corticosteroids for intubated patients have yielded conflicting results due to differences in the number of doses, types of corticosteroids, and timing and methods of administration to adult patients.

In our clinical practice, the planned extubation was usually performed four hour afterthe first injection of multiple prophylactic doses of dexamethasone. Sometimes, due to unpredictable conditions, critically ill patients need to delay the planned extubation after steroid treatment. Previous studies reported that most high-risk patients susceptible to postextubation upper air-way edema who fail extubation require reintubation within 48 to 72 hours^3^,^23^. However, little is known aboutthe after-effect of multiple-dose dexamethasone to prevent post extubation stridor.

The present study aims to determine the role of intravenous dexamethasone in preventing postextubation laryngeal edemalstridor in adults and children who were undergoing their first elective extubation in an ICU. The specific objectives of our study were to determine whether multiple doses of dexamethasone are effective to reduce or prevent postextubation airway obstruction and to investigate whether an after-effect (that is, a transient lingering benefit) exists 24 hours afterthe dis-continuation of dexamethasone.

## Methods

A total of 120 pediatric and adult patients who were admitted to the medical ICU between Jan 2003 to Feb 2006 were included in the study as approved by our local ethical committee. This was prospective, randomized, double blind, placebo-controlled study. After obtaining informed consent from the patients ortheir relatives, stratified random sampling was employed to select adults and children in 1:1 ratio among patients fulfilling eligibility criteria. The patients were then randomized in each group separately to receive either Dexamethas one or Placebo. The procedure adopted was permuted block randomization in orderto keep equal numbers in each group.

The patients who were on ventilators (Puritan bennett 840) for more than 24 hours were studied in ICU. All intubations were performed by a qualified experienced Anaesthetist in the operating room or in Accidental Emergency. Trachea was intubated with the standard endotracheal tube size according to their age. Children less than 10 yrs were intubated with uncuffed endotracheal tube. All patients requiring prolonged intubation and mechanical ventilation were sedated or paralyzed according to individual need to prevent agitation and excessive movement of the endotracheal tube, or interference with the ventilator. Routine nursing care included ETT suctioning every two hours and as needed to maintain a patent airway. The admitting diagnosis ranged from postoperative recovery to trauma.

### Exclusion Criteria:-

Following patients were not included in our study.

1. Patients having upper airway disease.
2. Patients who had undergone neck surgery.
3. Any anatomical deformity of upper airways
4. Patients already on steroids.
5. A history of extubation during the same hospitalization

In our study, patients were randomly assigned to two groups:-Study group and Control group.

1. Study Group comprising 60 patients with 30 adults and 30 children. In adults dexamethasone 8mg bolus i.v was given 4 hours prior to planned extubation, at extubation and at 6 and 12 hours after extubation.

In children, dexamethasone 0.5 mg kg^−1^ i.v with maximum of 8mg was given at similar intervals.

2. Control Group:-comprising 30 adults and 30 children who received placebo or saline at similar intervals.

The dexamethasone and the placebo were prepared in identical volume and labeled as A and B in a syringe to ensure administration in double blind fashion; neither the intensivist orderingthe drugs nor the person administering them was aware of the drugs being given to the patient till the end of the study.

Endotracheal extubations were followed according to standard ICU weaning protocol (ventilators used were Puritan Bennett 840 touch screen) as under: -

- Temperature of less than or equal to 38°C for more than eight hours,
- Discontinuous use of sedatives,
- Heart rate of more than or equal to 70 beats per minute and less than or equal to 130 beats per minute,
- Systolic blood pressure (SBP) of more than or equal to 80 mm Hg in the absence of vasopressor,
- Fraction of inspired oxygen (FiO_2_)of less than or equal to 60%, partial pressure of oxygen (PaO_2_) of more than or equal to 60 mm Hg, and PaO_2_/FiO_2_ ratio of more than 200,
- Positive end-expiratory pressure (PEEP) of less than or equal to 5 cm H_2_O,
- Respiratory rate = < 40/min
- Minute ventilation of less than or equal to 15 liters perminute, and
- pH of more than or equal to 7.3.
- Static Compliance (CST)> 33ml/cmH_2_O

Supplemental oxygen was continued to maintain an oxygen saturation of more than 95% as measured by a pulse oximeter.

All patients were clinically assessed for stridor, laryngeal edema (postextubation obstruction) after extubation forup to 24 hrs. The person assessing the parameters was unaware of the drug the patient had received. The patients with respiratory distress were assigned to take non-invasive positive-pressure ventilation (bi‐level positive airway pressure Puritan Bennett USA) by face mask if they failed in response to two doses of epinephrine inhalation and exhibited at least two of the following criteria of respiratory distress:

- Respiratory acidosis (defined as an arterial pH of less than 7.35 with a partial pressure of arterial carbon dioxide of more than 45 mm Hg),
- Clinical signs suggestive of respiratoty-muscle fatigue or increased respiratory effort (that is, use of accessory muscles, intercostal retraction, or paradoxical motion of the abdomen),
- Arespiratoty rate of more than 25 breaths per minute for two consecutive hours, and
- Hypoxemia (defined as an arterial oxygen saturation of less than 90%ora PaO_2_ of less than 80mmHg with a FiO_2_ of more than 50%.

Patients were reintubated with mechanical ventilation support if they met at least one of the following criteria:

- A pH of less than 7.3 with a partial pressure of carbon dioxide increase of more than 15mm Hg,
- A change in mental status rendering the patient unable to tolerate non-invasive ventilation,
- A decrease in the oxygen saturation to less than 85% despite the use ofa high FiO_2_ (a PaO_2_ of less than 50 mm Hg with an FiO_2_ of more than 70%),
- Lack of improvement in signs of respiratory-muscle fatigue,
- Hypotension with an SBP of less than 80 mm Hg form ore than 30 minutes despite adequate volume challenge,
- A diastolic blood pressure drop of more than 20 mm Hg, or
- Copious secretions that could not be cleared adequately or that were associated with acidosis, hypoxemia, or changes in mental status (somnolence, agitation, or diaphoresis).

Assessment of Airway status in adults and children was done as (Deming and Oech^19^):-

1. Laryngeal Dyspnea* Present/Absent
2. Laryngeal Stridor** Present/Absent
3. Laryngeal Edema Present/Absent

Evidenced during Laryngoscopy (Laryngoscopy is the gold standard method for diagnosing Postextubation Laryngeal Edema^24^)

* Laryngeal Dyspnea defined as occurrence of signs of upper airway obstruction i.e., prolonged Inspiratory phase associated with recruitment of accessory respiratory muscles.

** Laryngeal stridor defined as crowing sound present with inspiration.

Scoring for stridor in children was done as:-

0. No stridor

1. Stridor while crying
2. Stridor at rest
3. Severe Biphasic chest retractions

### Statistical analysis

Baseline characteristics were compared in both groups; ascertain comparability by using appropriate statistical tests. Chi-square test (χ^2^) and Fisher's exact ‘t’ test were used to evaluate difference in categorical data while unpaired ‘t’ test was used to evaluate statistical significance in mean values.

All analysis was performed by using computer software SPSS ver. 10.0forwindows and p-value of <0.05 was considered of statistical significance.

## Results

Following observations were made in our study:-

1. There was no statistically significant difference (p>0.05) among the groups in respect of age, sex, admitting diagnosis, ICU stay, intubation type and duration of intubation.(Table 1,2,3,4)

**Table 1:** Demographic data[(mean±S.D, n(%))]

Groups	Age(years)	Sex	
		Male	Female
**Study group**			
Adults	32.00±13.03	19(63.3)	11(36.6)
Children	7.87±3.85	16(53.33)	14(46.67)
**Control group**			
Adults	33.80±15.16	13(43.33)	17(56.67)
Children	7.70±3.93	22(73.33)	8(26.67)

**Table 2:** ICU stay comparison(days)

Group	Range	Mean±S.D
**Study group**		
Adults	3-10	5.70±2.25
Children	2–12	5.90±2.76
**Control group**		
Adults	2–12	5.20±2.05
children	3-12	5.43±2.01

**Table 3:** Comparison of admitting diagnosis between two groups in adults, n(%).

Diagnosis	Dexamethasone Group	Control Group
**In adults**		
1.Drug over dose	2(6.67)	1(3.33)
2.Organophosphorous Poisoning	11(36.67)	11(36.67)
3.Head injury	10(33.33)	6(20.00)
4.Cardiac surgery	2(6.67)	7(23.33)
5.Abdominal surgery	1(3.33)	3(10.00)
6.Miscellaneous	4(13.33)	2(6.67)
**In children**		
1.Organophosphorous Poisoning	4(13.33)	1(3.33)
2.Headinjury	13(43.33)	15(50.00)
3.Cardiac surgery	4(13.33)	7(23.33)
4.Neurological disease	3(10.00)	2(6.67)
5.Miscellaneous	6(20.00)	5(16.67)

**Table 4:** Comparison for duration of intubation in adults

Group	Duration of intubation	Number of cases (%)
**Study group**		
Adults	<72 hours	18(60.00)
	>72 hours	12(40.00)
Children	<72 hours	18(60.00)
	>72 hours	12(40.00)
**Control group**		
Adults	<72 hours	16(53.33)
	>72 hours	14(46.67)
Children	<72 hours	15(50.00)
	>72 hours	15(50.00)

2. There was statistically significantdifference (p =0.019) in comparison of failed extubation in children with respect to adults. In Dexamethasone group, in children 30% cases were of failed extubation while in control group, it was 63.33%.(Table 5).

**Table 5:** Comparison of failed extubation(n)

		Study group (Dexamethasone)	Control Gr. (Saline)	p value
Adults	Failed extubation	14	17	0.60
	Successful Extubation	16	13	
Children	Failed Extubation	9	19	0.019
	Successful Extubation	21	11	

3. On comparison between duration of intubation with incidence of failed extubation in adults in Dexa group, we found that with duration of intubation > 72 hours, failed extubation incidence was high with statistical significance (*p*=0.014). Similarly in Control group, incidence of failed extubation with respect to duration of intubation was statistically significant (*p* = 0.028). In children, in Dexamethasone group, the incidence of failed extubation with duration of intubation less than 72 hours was 22.22% while duration of intubation more than 72 hours was 41.67%. This difference although more with longer duration of intubation (more than 72 hours) but was statistically insignificant. (Table 6)

**Table 6:** Duration of intubation with respect to incidence of failed extubation

Group	Duration of intubation	Number of cases (%)	Failed extubation(%)	*p*-value
**Study group**				
Adults	<72 hours	18(60.00)	5(27.78)	0.014
	>72 hours	12(40.00)	9(75.00)	
Children	<72 hours	18(60.00)	4(22.22)	0.231
	>72 hours	12(40.00)	5(41.67)	
**Controlgroup**				
Adults	<72 hours	16(53.33)	6(37.50)	0.028
	>72 hours	14(46.67)	11(78.57.)	
Children	<72 hours	15(50.00)	8(53.33)	0.225
	>72 hours	15(50.00)	11(73.33)	

4. There was statistically significant variation on comparison of failed extubation/laryngeal edema incidence with that of sex. In Dexamethasone group, in adults 72.73%females had laryngeal edemawhich was statistically significant (p = 0.035). While in Control group, 70.59% females had failed extubation/ laryngeal edema. Although females had significant high incidence of failed extubation/laryngeal edema in Control group but did not reach statistical significance. (Table 7).

**Table 7:** Comparison of failed extubation / laryngeal edema with respect to sex in Adults

Group	Sex	Total cases	Failed extubation %	Test statistics	p-value	Remarks
Dexamethasone group						
Control group	Female	11	8(72.73)	Fisher's exact test	0.035	S
	Male	19	6(31.58)			
Control group	Female	17	12(70.59)		0.082	NS
	Male	13	5(38.46)			

5. Only in children between two groups, there was highly statistical significance in Airway assessment status (p = 0.004). Laryngeal edema was more in Control group (63.33%) in children as compared to Dexamethasone group (26.67%). While in adult patients there was an equivocal response in incidence of failed extubation/laryngeal edema between two groups. (Table 8)

**Table 8:** Airway assessment status in adults and children n(%)

Presence of Laryngeal stridor/Dexamethasone		Control	Test statistics	p-value
Dyspnea/Edema	Group	Group		
Adults	14/30(46.67)	17/30(56.67)	χ^2^=.601	0.438
**Children)**				
Present	8/30(26.67)	11/30(36.67)	χ^2^=8.15	0.004

## Discussion

The potential benefit of steroids to laryngeal edema is presumably based on its anti-inflammatory actions, which inhibit the release of inflammatory mediators and decrease capillary permeability. The risk of harm from steroid therapy for 24 hours or less to prevent postextubation laryngeal edema is negligible^21^‐^23^ The extent of the effect of prophylactic steroids on airway obstruction is still a matter of some controversy.

We conducted this randomized clinical trial to evaluate the effects of prophylactic dexamethasone therapy in preventing latyngealedema for adult patients and children in ICU setting. The choice of dexamethasone was based on its high anti-inflammatory potency, negligible mineral corticoid effects at therapeutic doses, and long duration of action^24^. We investigated the after-effect until 24 hours after the last dose of dexamethasone.

The incidence of laryngeal edema/stridorin our study, after extubation patient population has been reported to range from 26.67—36.67% which are not comparable to reports in literature ranging from 3-22%.^1^,^10^ This higher incidence of latyngeal edema and stridor is found to be associated with by many authors^2^,^3^,^7^ These results in literature, however, were obtained in studies evaluating short temi operative intubations; these studies did not include patients with wide spectrum of diagnosis & prolonged period ofintubation as is practiced in our study.

The increased incidence of laryngeal edema may be due to the fact that our study intended to select patients who required longer duration of intubation more than 24 hours and because of logistic concerns (4 doses of dexamethasone).

Our findings of steroid effect in reducing postextubation stridor were consistent with the results of the two recent studies in adult medical and surgical ICU settings^21^,^22^. In a recent study with a large number of subjects, Francois and colleagues^22^ reported that four doses of 20 mg of methylprednisolone, given at four hour intervals, significantly reduced the incidence of postextubation stridor from 22% to 3% and reduced the incidence of reintubation from 8% to 4%. However, the subjects in this study were not restricted to the high-risk patients for postextubation laryngeal edema (unlike the subjects in our study). The reduction of postextubation stridor in ourstudy was statistically significant, but not as dramatic as that of the study of Cheng and colleagues^21^. Another reason for this discrepancy may be the difference in the timing of extubation. Cheng and colleagues executed the extubation one hour after methylprednisolone administration over the span of 24 hours. The after-effect of dexamethasone validates the reduced incidence of postextubation stridor after multiple doses of dexamethasone.

The development of stridor is unpredictable and this type of acute upper airway obstruction can be characterized by range of symptoms ranging from hoarseness to severe obstruction requiring reintubation. Moreover, female sex is also risk factor for laryngeal edema identified in our study. Several authors have also stressed female gender as risk factor.^11^‐^13^. Our study results are in agreement with studies^1^,^10^,^14^‐^16^ who concluded that postextubation stridor was reduced in children with prophylactic administration of dexamethasone, but in adults, it did not appearto reduce the need for reintubation.

Our results are contradictory to the studies done by Gauss orgues P^11^, Courtney^17^, Tellez D l8 who demonstrated that incidence of laryngeal edema/stridor was not modified by corticosteroids (Dexamethasone).

Our study have substantiated that corticosteroids confer the benefits in children at high risk for postextubation laryngeal edema, whereas aroutine prophylactic use of corticosteroids to prevent postextubation stridorin every intubated patient is unwarranted. Dexamethasone and other steroids, in appropriate doses, can be helpful in alleviating laryngeal edema in intubated high-risk patients susceptible to air-way obstruction, such as those requiring repeated or prolonged intubations.

Based on this information, clinicians should consider initiating prophylactic corticosteroid therapy in this population. However; further studies are needed to establish the optimal dosing regimens as well as the sub-groups of patients at high risk forp ostextubation laryngeal edema who will derive the greatest benefit from this preventive steroid therapy.
